# The Recurrent E-Cadherin (CDH1) Mutation c.760G>A Causes Orofacial Clefts but Does Not Predispose to Hereditary Cancer

**DOI:** 10.3390/genes15111475

**Published:** 2024-11-15

**Authors:** Lea Gossner, Dietmar Rieder, Thomas Müller, Andreas R. Janecke

**Affiliations:** 1 Department of Pediatrics I, Medical University of Innsbruck, 6020 Innsbruck, Austria; lea.gossner@yahoo.de (L.G.); thomas.mueller@tirol-kliniken.at (T.M.); 2 Division of Bioinformatics, Medical University of Innsbruck, 6020 Innsbruck, Austria; dietmar.rieder@i-med.ac.at; 3 Institute of Human Genetics, Medical University of Innsbruck, 6020 Innsbruck, Austria

**Keywords:** E-cadherin, *CDH1* mutation, *CDH1* variant, cleft lip, cleft palate, genotype-phenotype correlation, dominant inheritance, *CDH1* germline mutation, syndromic CL/P

## Abstract

**Objective**: Congenital, non-syndromic orofacial clefts (CL/P) are infrequently monogenic in etiology. However, heterozygous pathogenic *CDH1* germline variants were reported in a few non-syndromic CL/P families, as well as in one syndromic form of CL/P: the blepharocheilodontic syndrome. *CDH1* encodes epithelial cadherin (E-cadherin), and close to 300 different pathogenic *CDH1* variants are listed in the ClinVar mutation database. The majority of *CDH1* germline variants are implicated in hereditary diffuse gastric cancer (HDGC) susceptibility. The purpose of this study was to classify the *CDH1* c.760G>A (p.Asp254Asn) mutation with respect to its resulting phenotype. **Methods**: Exome sequencing and targeted Sanger sequencing were performed in a family segregating CL/P. A review of pathogenic *CDH1* variants in ClinVar and those identified in a PubMed/MEDLINE search was performed. **Results**: We identified a family with six individuals, who were 35–77 years old (mean 56 years) at their last examination, uniformly affected with bilateral CL/P. The *CDH1* c.760G>A variant segregated with CL/P. This variant had been reported in 21 individuals, most often children and young adults, from six families. We determined a significant sex preponderance for this variant regarding CL/P: all 16 male and 5 of 11 female heterozygotes presented with CL/P. Furthermore, none of the heterozygous individuals in seven families reported any gastrointestinal tumors. **Conclusions**: The recurrent *CDH1* c.760G>A mutation confers a high risk for CL/P, with strong male preponderance. This review of 27 mutation carriers, including 3 who were 68, 70, and 77 years of age, indicates that c.760G>A does not confer an increased risk for HDGC. The relevance of differentiating craniofacial from cancer phenotypes in mutation carriers is substantial for precision medicine and for counseling families.

## 1. Introduction

Orofacial clefts (CL/P) comprise an isolated cleft palate and cleft lip with or without a cleft palate and can result from genetic and environmental risk factors. The incidence of CL/P within different populations is considered in the range of 1:700–1:1000 newborns. Affected individuals may experience feeding, hearing, speech, and psychological problems. Around 30% of CL/P cases are syndromic, and syndromic forms of CL/P frequently represent monogenic disorders, such as blepharocheilodontic syndrome (BCDS) [1]. Non-syndromic CL/P is infrequently monogenic [2], but heterozygous pathogenic *CDH1* germline variants were identified in a few non-syndromic CL/P families [3,4,5,6], as well as in BCDS [7,8]. *CDH1* encodes epithelial cadherin (E-cadherin), and close to 300 different pathogenic variants in *CDH1* are listed in the ClinVar mutation database (https://www.ncbi.nlm.nih.gov/clinvar/, accessed on 12 September 2024). The majority of *CDH1* germline variants were implicated in hereditary diffuse gastric cancer (HDGC) susceptibility [9,10,11,12]. However, 27 distinct pathogenic variants were implicated in CL/P, and eight distinct *CDH1* mutations were segregated with both CL/P and HDGC within families [5].

Although diffuse gastric cancer is the dominant tumor type in HDGC, other malignant neoplasms are common [5]. The incomplete penetrance and the phenotypic consequences of pathogenic *CDH1* mutations create significant challenges for genetic counseling and tumor prevention [9,12,13].

We report here the segregation of a recurrent c.760G>A in *CDH1* with CL/P in six adults from one family. These data and a literature review of 21 reported individuals with this genotype indicate that this particular variant predisposes to CL/P with high penetrance and with sex-dependent penetrance, but that it might not confer an elevated risk for HDGC.

## 2. Materials and Methods

The study was approved by the Institutional Review Boards of the Medical University of Innsbruck (No. UN4501), Innsbruck, Austria. Written informed consent was obtained from the patients for publication of this study and any accompanying images.

Genomic DNA was extracted from peripheral blood samples of 10 participating family members using standard procedures. The proband’s coding DNA fragments were enriched with the SureSelect Human All Exon V6 (61 Mb) kit (Agilent, Santa Clara, CA, USA) according to the protocol of the manufacturer and captured libraries were sequenced on a HiSeq platform (Illumina, San Diego, CA, USA) with 100 bp read length and paired-end sequencing. Reads were aligned to reference genome “Genome Reference Consortium Human Build 38 Organism: Homo sapiens (GRCh38)” (University of California Santa Clara, Santa Clara, CA, USA) and variants were called with the Genome Analysis Toolkit (GATK) version 4.0 (https://github.com/broadinstitute/gatk, accessed on 20 July 2020). Sequencing reads were also aligned to the human reference genome “Genome Reference Consortium Human Build 37 Organism: Homo sapiens (GRCh37)” with Burrows–Wheeler Alignment BWA-MEM [14] and variants were called with samtools [15]. The resulting lists of sequence variants were filtered for an allele frequency below 0.001, and for a predicted effect on protein function (missense, stop gain, frameshift, inframe insertion and deletion, canonical splice-site alteration). Pathogenic or likely pathogenic variants were preferentially searched for in genes associated with those listed in the gene panel “Clefting (Version 6.0)” (https://panelapp.genomicsengland.co.uk/panels/81/, accessed in February 2022). All variants were evaluated in silico for pathogenicity using CADD (http://cadd.gs.washington.edu/score, accessed in February 2022) [16]; missense variants were evaluated by PolyPhen-2 (http://genetics.bwh.harvard.edu/pph2, accessed in February 2022) [17] and SIFT (accessed in February 2022) [18]; and splice site variants were evaluated using SpliceAI lookup (https://spliceailookup.broadinstitute.org/, accessed in February 2022). ES data were evaluated for copy-number variants (CNVs), including single exon deletions and duplications with the panelcn.MOPS v1.28.0 software [19]. An analysis for CNVs, i.e., to detect partial and complete gene deletions or duplications, was complemented with chromosomal microarray analysis (Illumina HumanCytoSNP-12v2 BeadChip SNP array with 300 k markers, Illumina, San Diego, CA, USA).

Sanger sequencing of a genomic PCR fragment of 368 bp in size, obtained with forward primer 5′gtaaaacgacggccagtCCTAGGAAGGTGTGGCAGC and reverse primer 5′caggaaacagctatgacCTGTCCGTAGGAAGGATCAGC permitted *CDH1* variant validation and segregation within the family. The sequencing made use of M13 tags to the primers (small letters in primer sequences). *CDH1* variant designation is based on National Center for Biotechnology Information reference sequence for *CDH1* transcript *NM_004360.5*.

In order to obtain information on *CDH1* sequence variants that were considered to underlie clinical symptoms of the HDGC spectrum and/or CL/P, a list of the *CDH1* variants listed as pathogenic and likely pathogenic in the ClinVar mutation database was obtained; the literature cited with these ClinVar mutations as well as mutation-specific information obtained with PubMed/MEDLINE searches were evaluated for genotype-phenotype correlations. The PubMed/MEDLINE search was conducted with the following keywords: CDH1 mutation, CDH1 variant, CDH1 germline mutation, E-cadherin mutation, CDH1 cleft palate.

## 3. Results

**Figure 1:**
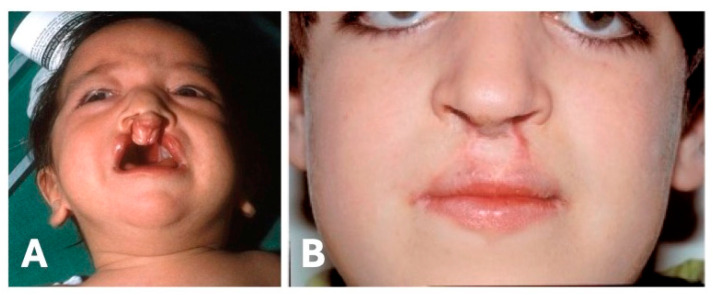
Typical familial expression of the CL/P phenotype before (**A**) and after (**B**) surgery.

We identified a Caucasian family with six individuals in three consecutive generations affected with bilateral, complete cleft lip and palate (Figure 1A,B). The orofacial clefts were expressed to the same degree of severity in all affected persons. The proband presented an additional unilateral congenital thumb hypoplasia, a rare component of BCDS. All family members were subjected to genetic counseling and detailed health histories were obtained that included inquiries to any known tumor history. None of the six individuals with CL/P (age range: 35–77 years, mean 56 years) had undergone a cancer check-up with endoscopy at this time. However, there was neither a history of tumors affecting the digestive tract, nor of those of the HDGC spectrum in mutation carriers and non-carriers in this family.

**Figure 2:**
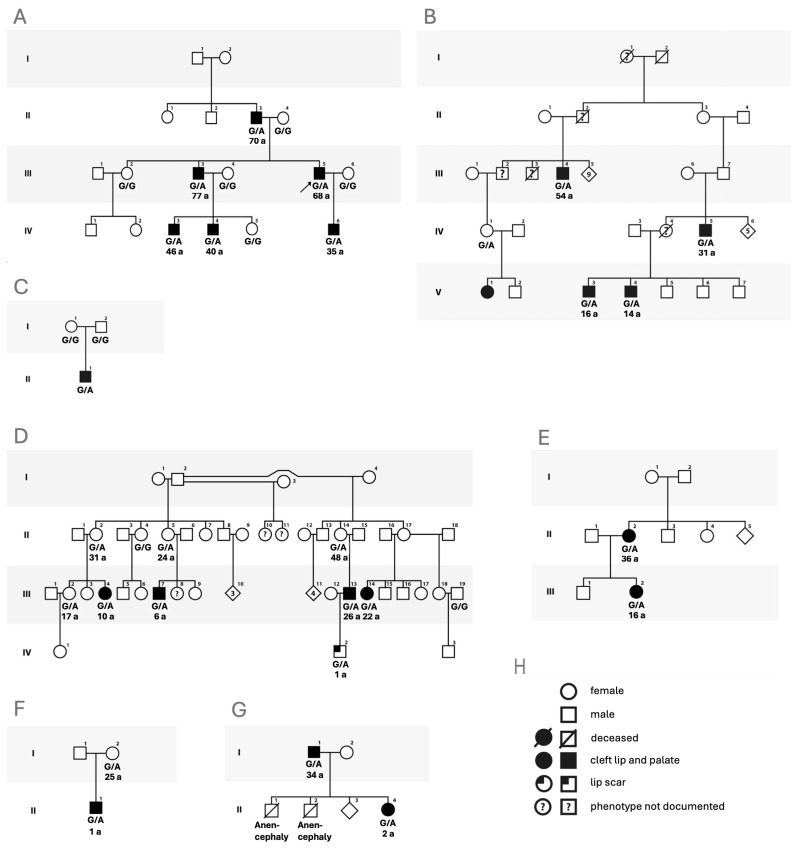
The CDH1 mutation c.760G>A underlies CL/P in 7 unrelated families. (**A**) The family studied here, the proband is denoted by an arrow. The status of CDH1 mutation c.760G>A is depicted below each genotyped family member’s symbol: G/G, wildtype; G/A: heterozygote, genotype is inferred in the founder II-3. Conclusive information on individuals in generation I regarding congenital CL/P could not be obtained. (**B**–**G**). Reported families with the CDH1 mutation c.760G>A segregating with CL/P. a, age at last examination (years). (**H**). Legend to symbols.

The c.760G>A mutation in *CDH1* was identified in the proband through exome sequencing and was shown to segregate with the phenotype in this family using targeted Sanger sequencing (Figure 2A).

This mutation is not listed in the population database gnomAD v4.1.0 (https://gnomad.broadinstitute.org/, accessed on 12 June 2024), indicating it is an ultrarare variant in the general population. However, in the literature referring to *CDH1* mutations and phenotypes, the c.760G>A mutation represents a frequently reported variant. The recurrent CDH1 variant c.760G>A was previously reported in 21 individuals from six families, of whom 15 individuals showed orofacial clefts (Figure 2B–G). Our review of the data indicates that the heterozygous c.760G>A mutation confers an overall risk of 77.8% (95%-CI: 0.5774–0.9138) for CL/P development. However, we find that this risk is significantly different between sexes: while all 16 male heterozygotes for this mutation presented with CL/P, 5 out of 11 female heterozygotes presented with CL/P (Fisher exact test statistic 0.0016, *p* < 0.05). The age at the last examination of the 27 reported heterozygotes for the CDH1 variant c.760G>A, with and without CL/P ranged from 1 to 77 years, with a mean and median age of 30 and 28 years. None of these heterozygous individuals reported any gastrointestinal tumors within or outside the HDGC spectrum.

## 4. Discussion

We identified a family with clustering of CL/P indicating autosomal-dominant inheritance. Formally, Y-chromosomal inheritance was another possibility, however unprecedented, and unlikely due to the small gene content of the Y chromosome. Genetic testing in this family followed the usual approach with ES, with special emphasis on the evaluation of known genes for non-syndromic and syndromic CL/P, and with the consideration of small sequence variants (single nucleotide variants, indels) and CNVs. CNV detection in ES data are generally complimented with chromosomal microarray analysis.

The vast majority of close to 300 reported that *CDH1* mutations are associated with HDGC and less commonly with other tumor susceptibility. A recent review of published *CDH1* mutations revealed 27 distinct germline *CDH1* mutations, including c.760G>A (p.Asp254Asn) [3,4,8], that are linked to congenital syndromic and non-syndromic orofacial clefts without apparent susceptibility to tumor development, whereas eight further *CDH1* mutations were associated with both HDGC and CL/P [5,9,10]. CL/P-only related mutations are predominantly missense variants at the conserved Asp254-Gln255-Asn-256-Asp257 ‘linker’ region and at other sites of CDH1-CDH1 binding as well as late-truncating variants [5,7]. This missense mutation causes the exchange of a highly conserved aspartic acid to asparagine at codon 254 in the CDH1 polypeptide (p.Asp254Asn). A detrimental effect of the CDH1 p.Asp254Asn mutation has been shown; its expression induced incomplete development to total absence of head structures in zebrafish embryos, and resulted in a decreased adherence of cells in a MCF7 cell model, supposedly by interfering with cis-dimerization of E-cadherin and the rigidity of the extracellular domain [8]. Mutations associated with the HDGC spectrum include almost all cadherin truncating variants (stop codons, frameshifts, splicing) suggesting a complete loss-of-function as a disease mechanism, and that HDGC-associated missense variants confer a loss-of-function, too [5]. The loss of the other allele and promoter methylation occur as second genetic hits in CDH1-related HDGC [20,21]. Particular CDH1 germline mutations that are associated with HDGC and CLP in families are predominantly splice-site mutations, such as the c.687+1G>A mutation, that leads to a protein with a 14 amino acid in-frame deletion, which might act simultaneously both by a complete loss of the normal function and HDGC and by expression of an abnormal protein that is expected to affect CDH1-CDH1 binding like linker missense mutations [6].

The relevance of differentiating craniofacial from cancer phenotypes is substantial in order to provide tumor preventive measures and for counseling couples on the risk of having a child with CL/P. Predicting the effect of individual *CDH1* mutations is complicated by incomplete and age-dependent penetrance with respect to HDGC as well as CL/P. Pathogenic *CDH1* variants were reported to convey a lifetime risk (until the age of 80 years) of diffuse gastric cancer in the range from 37% to 70% in men and 25% to 83% in women; the average age of gastric cancer diagnosis ranged from 38 to 80 years, with the earliest reported diagnosis made at age 14 years [10,11,22,23,24]. Here, we provide evidence for classification of the *CDH1* mutation c.760G>A as causing CL/P-only, by reporting its segregation with CL/P in a large family with an average age of mutation carriers of 56 years. In total, 27 heterozygous individuals with this mutation are now known, of whom 21 presented with CL/P. None of these individuals, including three individuals of 68, 70, and 77 years of age reported here, presented with HDGC, and this holds true for one family with two heterozygous individuals for a c.760G>T mutation affecting the same codon (p.Asp254Tyr) [7]. However, we cannot exclude the later occurrence of gastric cancer and lobular breast cancer in c.760G>A carriers in the family reported here; however, the current absence in all 27 mutation carriers from seven families appears noticeable to us. It is a limitation of this study that all c.760G>A carriers are, or were, at an age that does not rule out the occurrence of CDH1-related tumor development.

Male preponderance for CL/P and female preponderance for CP in general has long been noticed. Male preponderance for CL/P development in c.760G>A carriers is significant but unexplained at present by the mutation itself. Of note, trio-based sex-stratified genome-wide association studies recently identified 13 loci that appear to contribute to inherent sex-specific risks [25]. Genetic risk factors might contribute to the phenotype independently or dependent on the presence of environmental risks for CL/P development such as exposure to maternal multivitamin use [26], maternal alcohol consumption [27] or smoking [28].

## 5. Conclusions

The recurrent *CDH1* c.760G>A mutation confers an overall risk of 78% for CL/P, with strong male preponderance. The identification of c.760G>A in three individuals of 68, 70, and 77 years with CL/P and without HDGC may indicate that it does not confer an increased risk for HDGC. The relevance of differentiating craniofacial from cancer phenotypes is substantial in order to provide tumor preventive measures [12,13] and for counseling couples on the risk of having a child with CL/P.

## Data Availability

All data relating to this study are contained in this report. The exome sequencing data set of the index patient is unavailable due to privacy restrictions.

## References

[1] Allanson J.E., McGillivray B.C. (1985). Fam. Clefting Syndr. Ectropion Dent. Anomaly—Without Limb Anomalies. Clin. Genet..

[2] Dixon M.J., Marazita M.L., Beaty T.H., Murray J.C. (2011). Cleft lip and palate: Understanding genetic and environmental influences. Nat. Rev. Genet..

[3] Brito L.A., Yamamoto G.L., Melo S., Malcher C., Ferreira S.G., Figueiredo J., Alvizi L., Kobayashi G.S., Naslavsky M.S., Alonso N. (2015). Rare Variants in the Epithelial Cadherin Gene Underlying the Genetic Etiology of Nonsyndromic Cleft Lip with or Without Cleft Palate. Hum. Mutat..

[4] Cox L.L., Cox T.C., Moreno Uribe L.M., Zhu Y., Richter C.T., Nidey N., Standley J.M., Deng M., Blue E., Chong J.X. (2018). Mutations in the Epithelial Cadherin-p120-Catenin Complex Cause Mendelian Non-Syndromic Cleft Lip with or Without Cleft Palate. Am. J. Hum. Genet..

[5] Selvanathan A., Nixon C.Y., Zhu Y., Scietti L., Forneris F., Uribe L.M.M., Lidral A.C., Jezewski P.A., Mulliken J.B., Murray J.C. (2020). CDH1 Mutation Distribution and Type Suggests Genetic Differences between the Etiology of Orofacial Clefting and Gastric Cancer. Genes.

[6] Obermair F., Rammer M., Burghofer J., Malli T., Schossig A., Wimmer K., Kranewitter W., Mayrbaeurl B., Duba H.C., Webersinke G. (2019). Cleft lip/palate and hereditary diffuse gastric cancer: Report of a family harboring a CDH1 c.687 + 1G > A germline mutation and review of the literature. Fam. Cancer.

[7] Ghoumid J., Stichelbout M., Jourdain A.S., Frenois F., Lejeune-Dumoulin S., Alex-Cordier M.P., Lebrun M., Guerreschi P., Duquennoy-Martinot V., Vinchon M. (2017). Blepharocheilodontic syndrome is a CDH1 pathway-related disorder due to mutations in CDH1 and CTNND1. Genet. Med..

[8] Kievit A., Tessadori F., Douben H., Jordens I., Maurice M., Hoogeboom J., Hennekam R., Nampoothiri S., Kayserili H., Castori M. (2018). Variants in members of the cadherin-catenin complex, CDH1 and CTNND1, cause blepharocheilodontic syndrome. Eur. J. Hum. Genet..

[9] Luo X., Maciaszek J.L., Thompson B.A., Leong H.S., Dixon K., Sousa S., Anderson M., Roberts M.E., Lee K., Spurdle A.B. (2023). Optimising clinical care through CDH1-specific germline variant curation: Improvement of clinical assertions and updated curation guidelines. J. Med. Genet..

[10] Lee K., Krempely K., Roberts M.E., Anderson M.J., Carneiro F., Chao E., Dixon K., Figueiredo J., Ghosh R., Huntsman D. (2018). Specifications of the ACMG/AMP variant curation guidelines for the analysis of germline CDH1 sequence variants. Hum. Mutat..

[11] Roberts M.E., Ranola J.M.O., Marshall M.L., Susswein L.R., Graceffo S., Bohnert K., Tsai G., Klein R.T., Hruska K.S., Shirts B.H. (2019). Comparison of CDH1 Penetrance Estimates in Clinically Ascertained Families vs Families Ascertained for Multiple Gastric Cancers. JAMA Oncol..

[12] Carneiro F. (2022). Familial and hereditary gastric cancer, an overview. Best. Pract. Res. Clin. Gastroenterol..

[13] Garcia-Pelaez J., Barbosa-Matos R., Lobo S., Dias A., Garrido L., Castedo S., Sousa S., Pinheiro H., Sousa L., Monteiro R. (2023). Genotype-first approach to identify associations between CDH1 germline variants and cancer phenotypes: A multicentre study by the European Reference Network on Genetic Tumour Risk Syndromes. Lancet Oncol..

[14] Li H. (2013). Aligning sequence reads, clone sequences and assembly contigs with BWA-MEM. arXiv.

[15] Li H., Handsaker B., Wysoker A., Fennell T., Ruan J., Homer N., Marth G., Abecasis G., Durbin R., 1000 Genome Project Data Processing Subgroup (2009). The Sequence Alignment/Map format and SAMtools. Bioinformatics.

[16] Rentzsch P., Witten D., Cooper G.M., Shendure J., Kircher M. (2019). CADD: Predicting the deleteriousness of variants throughout the human genome. Nucleic Acids Res..

[17] Adzhubei I.A., Schmidt S., Peshkin L., Ramensky V.E., Gerasimova A., Bork P., Kondrashov A.S., Sunyaev S.R. (2010). A method and server for predicting damaging missense mutations. Nat. Methods.

[18] Ng P.C., Henikoff S. (2003). SIFT: Predicting amino acid changes that affect protein function. Nucleic Acids Res..

[19] Povysil G., Tzika A., Vogt J., Haunschmid V., Messiaen L., Zschocke J., Klambauer G., Hochreiter S., Wimmer K. (2017). panelcn.MOPS: Copy-number detection in targeted NGS panel data for clinical diagnostics. Hum. Mutat..

[20] Guilford P.J., Hopkins J.B., Grady W.M., Markowitz S.D., Willis J., Lynch H., Rajput A., Wiesner G.L., Lindor N.M., Burgart L.J. (1999). E-cadherin germline mutations define an inherited cancer syndrome dominated by diffuse gastric cancer. Hum. Mutat..

[21] Grady W.M., Willis J., Guilford P.J., Dunbier A.K., Toro T.T., Lynch H., Wiesner G., Ferguson K., Eng C., Park J.G. (2000). Methylation of the CDH1 promoter as the second genetic hit in hereditary diffuse gastric cancer. Nat. Genet..

[22] Pharoah P.D., Guilford P., Caldas C., International Gastric Cancer Linkage Consortium (2001). Incidence of gastric cancer and breast cancer in CDH1 (E-cadherin) mutation carriers from hereditary diffuse gastric cancer families. Gastroenterology.

[23] Xicola R.M., Li S., Rodriguez N., Reinecke P., Karam R., Speare V., Black M.H., LaDuca H., Llor X. (2019). Clinical features and cancer risk in families with pathogenic CDH1 variants irrespective of clinical criteria. J. Med. Genet..

[24] Lerner B.A., Xicola R.M., Rodriguez N.J., Karam R., Llor X. (2023). Simplified and more sensitive criteria for identifying individuals with pathogenic CDH1 variants. J. Med. Genet..

[25] Robinson K., Parrish R., Adeyemo W.L., Beaty T.H., Butali A., Buxo C.J., Gowans L.J.J., Hecht J.T., Moreno Uribe L., Murray J.C. (2024). Genome-wide study of gene-by-sex interactions identifies risks for cleft palate. Hum. Genet..

[26] Yoshida S., Takeuchi M., Kawakami C., Kawakami K., Ito S., Japan Environment and Children’s Study Group (2020). Maternal multivitamin intake and orofacial clefts in offspring: Japan Environment and Children’s Study (JECS) cohort study. BMJ Open.

[27] DeRoo L.A., Wilcox A.J., Lie R.T., Romitti P.A., Pedersen D.A., Munger R.G., Moreno Uribe L.M., Wehby G.L. (2016). Maternal alcohol binge-drinking in the first trimester and the risk of orofacial clefts in offspring: A large population-based pooling study. Eur. J. Epidemiol..

[28] Kummet C.M., Moreno L.M., Wilcox A.J., Romitti P.A., DeRoo L.A., Munger R.G., Lie R.T., Wehby G.L. (2016). Passive Smoke Exposure as a Risk Factor for Oral Clefts-A Large International Population-Based Study. Am. J. Epidemiol..

